# Protocol for a randomised controlled feasibility study examining the efficacy of brief cognitive therapy for the treatment of panic disorder in adolescents (PANDA)

**DOI:** 10.1186/s40814-022-01009-z

**Published:** 2022-03-03

**Authors:** Polly Waite

**Affiliations:** 1 School of Psychology and Clinical Language Sciences, University of Reading, Reading, RG6 6AL UK; 2 Departments of Experimental Psychology and Psychiatry, University of Oxford, Oxford, OX2 6GG UK

**Keywords:** Panic disorder, Adolescent, Young people, Youth, Cognitive therapy, CBT, Psychological treatment, Brief

## Abstract

**Background:**

Panic disorder occurs in between 1 and 3% of adolescents, is associated with high levels of co-morbidity, and without treatment, appears to have a chronic course. To improve access to effective psychological interventions, briefer versions of cognitive behaviour therapy (CBT) have been developed and evaluated for preadolescent children with anxiety disorders. However, there are currently no brief evidence-based CBT interventions for adolescents with anxiety disorders that can be delivered in less than eight sessions. Given that a brief version of cognitive therapy has been shown to be effective in adults with panic disorder, it is possible that an adapted version could be effective for adolescents with panic disorder.

**Methods:**

The study will examine whether a definitive trial can be conducted, based on a single-centre feasibility randomised controlled trial using several well-defined criteria. Between 30 and 48 young people (age 11–18 years) who meet diagnostic criteria for panic disorder, attending a routine clinical service will be randomly allocated to receive either (i) brief cognitive therapy or (ii) a general form of CBT treatment that is more commonly used for adolescents with anxiety disorders. Both will be delivered 1:1 by a therapist and involve five treatment sessions and two booster sessions. Young people’s outcomes will be assessed at the end of treatment and at 3-month follow-up, and qualitative interviews will be conducted to examine acceptability. We will also explore outcomes 1 year after the completion of treatment.

**Discussion:**

This study will test the feasibility of a randomised controlled trial to compare brief cognitive therapy to a general form of CBT for adolescents with panic disorder in the UK. The outputs from the study will provide a clear indication of the feasibility of a future definitive trial and, if indicated, the critical resources that will be required and key information to inform the design and maximise the successful completion of the trial. This has the potential to bring direct benefits to young people and their families, as well as services and society more broadly.

**Trial registration:**

This trial is registered on the ISRCTN Registry, registration number ISRCTN14884288, registered retrospectively on 05/12/2019.

**Supplementary Information:**

The online version contains supplementary material available at 10.1186/s40814-022-01009-z.

## Introduction

For many people, panic disorder begins in adolescence [1], with between 1 and 3% of adolescents aged 11–19 years meeting diagnostic criteria for panic disorder [2, 3]. This early onset may be associated with more severe symptoms and a worse outcome than panic disorder that starts in adulthood [4]. Young people with panic disorder commonly avoid a range of environments, including school cafeterias or restaurants, small rooms and auditoriums [5], which is likely to have a significant impact on functioning at school and with friends. Furthermore, panic disorder in adolescence has high rates of comorbidity, including other anxiety disorders and depression, and is associated with the subsequent onset and persistence of alcohol abuse and dependence [5–7]. If left untreated, it appears to have a chronic course [6]. These factors highlight the need for effective and accessible treatments.

Two psychological treatments have been extensively evaluated with adults with panic disorder and shown to be similarly effective in their original format (i.e. 12 to 15 sessions): panic control treatment (PCT) [8] and cognitive therapy (CT) for panic disorder [9]. Both treatments have been shown to be superior to a range of other treatments including relaxation therapy, supportive psychotherapy and medication [10]. However, when briefer forms of the two treatments were developed and evaluated, only the brief form of CT remained as effective as the full treatment [9, 11]. Specifically, a version of CT involving self-study modules and 5 sessions (plus two booster sessions) of therapy (B-CT) did not differ significantly from the full 12 session (plus two booster sessions) treatments (F-CT) immediately or at 12-month follow-up (panic-free at post-treatment: F-CT 79%, B-CT 71%; 12-month follow-up: F-CT 71%, B-CT 79%). No participants dropped out of treatment, and there were large effect sizes for both the full and brief treatments on a measure of panic symptoms from pre- to post-treatment and pre- to 12-month follow-up (post-treatment: F-CT *d* = 2.9, B-CT *d* = 2.9; 12-month follow-up: F-CT *d* = 2.8, B-CT *d* = 3.2). Both treatments were also significantly more effective than a waitlist control in reducing panic symptoms on a range of measures (*d* ranged from 1.35 to 2.98).

In contrast to treatment for adults, the treatment of panic disorder in adolescents has been largely neglected, and a considerable number of randomised controlled trials treating anxiety in children and adolescents have excluded young people who have panic disorder as the primary problem [12–14]. The most substantial evidence base for CBT for a broad range of anxiety disorders in children and adolescents comes from trials using a general, transdiagnostic treatment approach (e.g. the ‘Coping Cat’ treatment protocol [15] or the adolescent version, the ‘C.A.T. Project’ [16]). The treatment involves anxiety management (e.g. psychoeducation, cognitive restructuring and relaxation techniques) prior to graded exposure, where the young person learns to face their fears through exposure to the feared situation or stimulus in a graded way. Meta-analyses have demonstrated that around 50% of children and adolescents are free of their primary diagnosis at the end of CBT [17]. However, outcomes for young people with panic disorder specifically are unknown. In the UK, CBT appears to be the most used treatment approach by clinicians in routine clinical practice to treat young people with panic disorder. However, a recent national survey of over 400 clinicians working in Child and Adolescent Mental Health Services (CAMHS) in the National Health Service (NHS) found that only 2.6% was able to identify any specific treatment protocols to treat young people with panic disorder, either within transdiagnostic or disorder-specific treatments [18].

Currently, young people face significant difficulties to access evidence-based treatments. Fewer than one in five adolescents in need of treatment receive appropriate psychological interventions [19], and many face significant delays or spend months on waiting lists for treatment within routine clinical services [20]. In order to improve access to effective psychological interventions, briefer versions of CBT have been developed that can be delivered by non-specialists, so that more intensive treatments can be reserved for those who do not, or who are unlikely to, benefit from a brief treatment [21]. Brief CBT has recently been defined as referring to either low intensity CBT (i.e. 6 h or less of contact time with a therapist, using self-help materials) and/or brief high intensity CBT (i.e. based on the standard evidence-based CBT treatment, with therapy contact time 50% or less than the full CBT intervention) [22]. Suitable brief CBT treatments have been developed and evaluated for preadolescent children [23]; however, there has been limited research attention on brief CBT interventions for adolescents with anxiety disorders. Of the sixteen studies of psychological therapies for adolescents with anxiety disorders (including panic disorder) identified in a recent meta-analysis [24], only one study [25], involving adolescents with a range of anxiety disorders, fulfilled the definition of brief CBT.

Many treatments for anxiety disorders in children and adolescents have successfully been adapted from treatments shown to be effective in adults. Although Barlow and Craske’s PCT has been successfully adapted for adolescents with panic disorder in the USA (PCT-A) [26], the two derivations of treatment require a considerable amount of therapist time (between 11 and 22 h of therapy) [27, 28]. In addition, the intensive form of treatment, which has the most empirical support, would be difficult to implement in NHS CAMHS settings as it requires treatment to be conducted by a therapist for several hours over consecutive days. To date, there has been no adaptation of Clark’s cognitive therapy for adolescents with panic disorder, despite emerging evidence that the psychological processes targeted in CT for adults may also be evident in adolescents [29–31]. Given that the brief version of this treatment has been shown to be effective in adults with panic disorder, with a large effect size, it is possible that recovery rates for adolescents could be substantially improved, and treatment considerably shortened by using this alternative treatment approach, adapted for use with adolescents.

To maximise the likelihood of a future successful large-scale randomised controlled trial (RCT), the aim of this study is to test the feasibility of a RCT to compare brief cognitive therapy for panic disorder (adapted for adolescents) to a general form of CBT treatment that is more commonly used for adolescents with anxiety disorders, including panic disorder, in the UK.

## Method

### Aims of the study

The specific aims of this study are as follows:

- Identify appropriate clinical outcome and economic measures for a subsequent definitive trial.
- Explore the acceptability of the treatments and trial procedures.
- Establish likely recruitment rates.
- Establish the likely rate of treatment drop-out.
- Establish likely retention to research assessments post treatment and at 3-month follow-up.
- Explore retention to a brief 12-month follow-up.
- Establish if brief cognitive therapy can be delivered so that it is clearly distinct from a general form of CBT, with high levels of fidelity by practitioners and credibility with patients in both arms.
- Conduct exploratory analyses of possible outcomes for the two treatments including changes in anxiety symptoms, diagnostic status, quality of life, healthcare resource use and other outcomes identified through patient and public involvement and engagement (PPIE).
- Describe negative impacts of the treatments and the trial procedures (to patients, their parents and clinicians).
- Explore young people’s outcomes on measures of symptom and functional impairment.

The outputs from the study will provide a clear indication of the feasibility of a future definitive trial and, if indicated, the critical resources that will be required and key information to inform the design and maximise the successful completion of the trial. Information about how and when each aim will be measured, and the criteria required to be met to warrant progression to a full trial, can be found in Table 1. Progression criteria were developed based on recent similar studies (e.g. [32]) and in consultation with PPIE representatives, clinicians and researchers. Recruitment, retention, distinctness of treatment and fidelity of treatment delivery will be evaluated and determined as meeting one of three success levels using the following traffic light system:

- Green indicates that progression to a definitive trial is possible without needing to substantially change design or delivery.
- Amber indicates a need for more resources and/or new ideas for recruiting and retaining participants, ensuring that treatments are distinct and delivered with high levels of fidelity.
- Red indicates that a definitive trial may not be viable.

**Table 1:** Aims, outcome measures and progression criteria

	Aims	Measure	Timepoint	Progression criteria
a.	Identify appropriate clinical outcome and economic measures for a subsequent definitive trial	Clinical outcomes: ADIS-C/P/KSADS-C/P (or DAWBA and ADIS-panic section) (diagnosis and CSR)	Routine assessment/pre-randomisation	Appropriate measures identified
		Clinical outcomes: PDSS-A, RCADS-C/P, CAIS-C/P Health economic measures: CSRI, EQ-5D, CHU-9D	Enrolment/pre-treatment	
		Clinical outcomes: PDSS-A, RCADS-C/P, CAIS-C/P Health economic measures: CSRI, EQ-5D, CHU-9D	Post-treatment	
		Clinical outcomes: ADIS-C/P/KSADS-C/P (or DAWBA & ADIS-panic section) (diagnosis and CSR), CGI-I, PDSS-A, RCADS-C/P, CAIS-C/P Health economic measures: CSRI, EQ-5D, CHU-9D	3-month follow-up	
b.	Explore the acceptability of the treatments and trial procedures	Credibility and expectation of improvement scale-C/P	Enrolment	No serious concerns raised
		ESQ-C/P	Post-treatment and 3-month follow-up	No serious concerns raised
		Qualitative interview (young people, parent/carers, clinicians)	Post-treatment/3-month follow-up	No serious concerns raised
c.	Establish likely recruitment rates	Screening logs maintained by site *n* = Approached *n*= Accepting *n* = Declining Reasons (coded) for decline of study invitation	At study entry and ongoing through study	Green: ≥ 30 participants recruited; ≥ 80% eligible participants agree to randomisation
				Amber: 20–29 participants recruited; 70–79% eligible participants agree to randomisation
				Red: < 20 young people are recruited; < 70% eligible participants agree to randomisation
d.	Establish the likely rate of treatment drop-out	Number withdrawing from treatment	Ongoing through study	Green: Treatment drop-out rate of ≤ 20% in both treatment arms
				Amber: Treatment drop-out rate of 21–30% in both treatment arms
				Red: Treatment drop-out rate of > 30% in both treatment arms
e.	Establish likely retention to research assessments post-treatment and at 3-month follow-up	Number of completed assessments - PDSS-A - All other clinical and health economic outcome measures	Ongoing through study	Green: ≥ 80% of participants will complete the PDSS-A at post-treatment and 3-month follow-up assessment
				Amber: 70–79% of participants complete the PDSS-A at post-treatment and 3-month follow-up assessment
				Red: < 70% of participants complete the PDSS-A at post-treatment and 3-month follow-up assessment
f.	Explore retention to a brief 12-month follow-up	Clinical outcomes: PDSS-A	12-months follow-up	No set criteria
g.	Establish if brief cognitive therapy can be delivered so that it is clearly distinct from a general form of CBT, with high levels of fidelity by practitioners in both arms	Therapy contents checklist	Each clinical session	Green: In both arms, sessions contain ≥ 80% ‘allowable’ features of the specific intervention
				Amber: In both arms, sessions contain 70–79% ‘allowable’ features of the specific intervention
				Red: In both arms, sessions contain < 70% ‘allowable’ features of the specific intervention
h.	Conduct exploratory analyses of possible outcomes for the two treatments including changes in anxiety symptoms, diagnostic status, quality of life and healthcare resource use	Clinical outcomes: PDSS-A, RCADS-C/P, CAIS-C/P Health economic measures: CSRI, EQ-5D, CHU-9D	Post-treatment	Not applicable
		Clinical outcomes: ADIS-C/P/KSADS-C/P (diagnosis and CSR), CGI-I, PDSS-A, RCADS-C/P, CAIS-C/P Health economic measures: CSRI, EQ-5D, CHU-9D	3-month follow-up	Not applicable
		Clinical outcomes: PDSS-A	12-months follow-up	Not applicable
i.	Describe negative impacts of the treatments and the trial procedures (to young people, their parents/carers and clinicians)	Adverse events	Ongoing through study	Serious negative impacts do not occur because of participation in the trial
		Qualitative interview (young people, parent/carers, clinicians)	Post-treatment/3-month follow-up	No serious impacts raised
j.	Explore young people’s outcomes on measures of symptom and functional impairment	Symptom measures: PDSS-A, RCADS-C/P, CAIS-C/P Functional impairment: ORS	Post-treatment	Not applicable
		Symptom measures: PDSS-A, RCADS-C/P, CAIS-C/P Functional impairment: ORS	3-month follow-up	Not applicable
		Clinical outcomes: PDSS-A	12-month follow-up	Not applicable

To feel confident that a definitive trial can be delivered without major study redesign, all progression criteria would need to be met (i.e. no serious concerns raised) or met within reasonable limits (i.e. within the green or amber traffic light domains).

#### Design

This study is a parallel design RCT comparing brief cognitive therapy to a general form of CBT treatment that is more commonly used for adolescents with anxiety disorders. It will be conducted within the Anxiety and Depression in Young people (AnDY) Research Clinic at the University of Reading. Young people and their parent/carer’s expectations of treatment will be assessed prior to the beginning of treatment using a brief questionnaire [33]. Additionally, qualitative interviews will be conducted with a subsample of participating young people post-treatment to explore their experience of treatment. We will also interview parents/carers and key stakeholders (e.g. service managers, study clinicians and referrers) about their experiences.

Young people, including service users (with panic disorder and/or other anxiety disorder), parents and stakeholders have been involved in all stages of the study. Young people have been involved in adapting the questionnaires and workbooks used to support cognitive therapy and advising on recruitment. Parents have been consulted on the development of an accompanying workbook for parents/carers for those in the cognitive therapy arm. Stakeholders, including teachers and GPs, have advised on recruitment and screening procedures.

#### Setting

Participants will be recruited to the study through the AnDY Research Clinic at the University of Reading, a clinical service that is funded by local NHS commissioning. The AnDY Research Clinic offers assessments, treatment and research to children and young people who are experiencing difficulties with anxiety and/or depression. Assessment and treatment sessions will be conducted either face to face within the clinic or via video-conferencing software, whenever restrictions due to the coronavirus pandemic prohibit face-to-face meetings.

#### Participants

Between 30 and 48 participants will be recruited to the feasibility study. To be included in the study, the young person must be aged 11–18 years at assessment and meet diagnostic criteria for panic disorder [34], as either the primary or secondary presenting disorder. They must have had at least one panic attack in the month prior to assessment. If the young person has a comorbid medical condition (such as asthma, epilepsy or cardiovascular disease), the young person’s GP must have been consulted and given the opinion that this will not interfere with treatment delivery. They must not be taking psychotropic medication, or alternatively, they must be willing to be withdrawn from medication before the start of the trial under the supervision of their GP. A minimum 6-week drug-free period for SSRIs and a 2-day drug-free period for benzodiazepines will be required before the young person can start treatment, and they must agree not to start medication during the trial. They must be able to speak English, willing to accept random allocation and engage in the treatment. Young people will be excluded if they have a co-morbid condition that is likely to interfere with treatment delivery, such as an established autistic spectrum disorder, learning disabilities, suicidal intent or recurrent or potentially life-limiting self-harm (i.e. current frequency of at least once per week or self-harm that requires medical attention); they been identified by social services as currently ‘at risk’ due to, for example, child protection concerns; or if they are receiving a psychological intervention or have received previous treatment with cognitive therapy or CBT for panic disorder.

#### Sample size

As is typical in feasibility studies, the sample size is not based upon a power calculation [35]. Across both groups, we will aim to recruit 48 participants but will accept a minimum of 30 participants. If adverse events or significant deterioration were likely to occur as a result of participating in the trial, this would be expected to be observable within this sample size, and there will be sufficient throughput of potential participants within the recruitment period to examine recruitment and retention rates and participant flow through the trial and to examine treatment integrity [36–38]. The proposed sample size is also sufficient to provide an estimate of the variation in outcomes on which to power a definitive trial, if indicated, based on continuous outcomes (i.e. panic disorder severity symptoms).

A subsample will be involved in qualitative interviews after the treatment has been delivered. We will use purposive sampling and sample for a diverse range of demographic and clinical characteristics. The adequacy of the sample size will be continuously evaluated during the study, and recruitment to the interviews will come to an end when the sample holds sufficient information power to develop new knowledge [39, 40]. However, it may also be shaped and constrained by the number of potential interviewees, particularly in the stakeholder sample, as well as time and resources available [40]. At this stage, it is anticipated that this is likely to involve around 10–15 young people, 10–15 parents, and 5–10 stakeholders.

#### Procedure

**Fig. 1:**
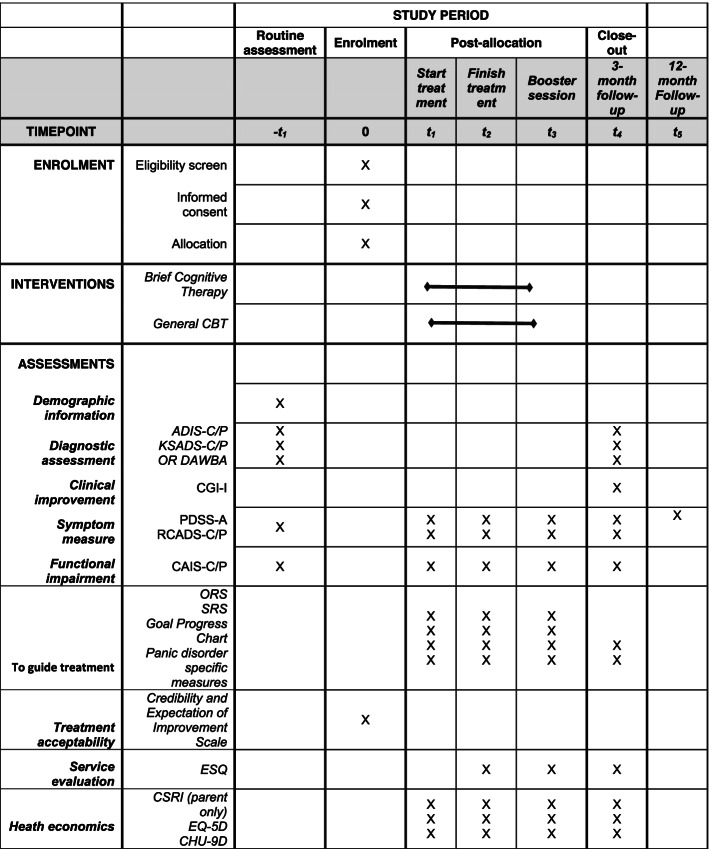
SPIRIT schedule of enrolment, interventions and assessments. ADIS-C/P, anxiety disorder interview schedule for DSM-IV child and parent version (anxiety section and common comorbid disorders). Panic disorder-specific measures: ACQ-adapted, Agoraphobia Cognitions Questionnaire (adapted); BSQ-adapted, Body Sensations Questionnaire (adapted); SBQ adapted, Safety Behaviour Questionnaire (adapted); MI adapted, mobility inventory (adapted); CAIS-C/P, Child Anxiety Impact Scale — child and parent version; CGI-I, Clinical Global Impression — Improvement; CHU-9D, Child Health Utility (paediatric quality of life); CSRI, Client Services Receipt Inventory; EQ-5D-Y, EuroQol (quality of life); ESQ, Experience of Service Questionnaire; KSADS-C/P, Kiddie Schedule for Affective Disorders and Schizophrenia — child and parent version (depression screen and supplement (including persistent depressive disorder), mania screen (supplement only if screening questions are endorsed)); ORS, Outcome Rating Scale; PDSS-A, Panic Disorder Severity Scale for Adolescents; RCADS-C/P, Revised Child Anxiety and Depression Scale-child and parent versions; SRS, Session Rating Scale

**Fig. 2:**
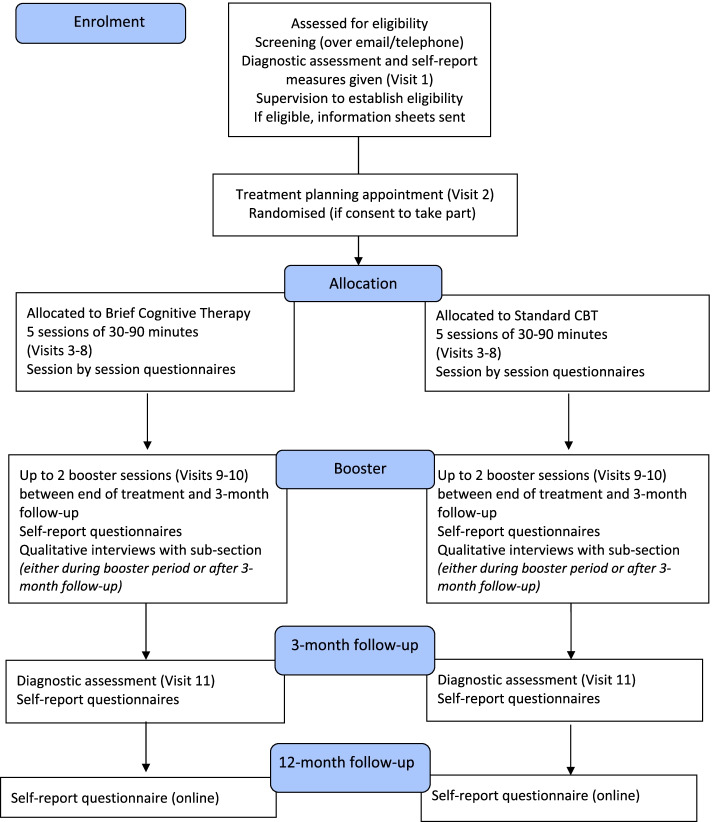
Overview of study procedure

The study procedure is in line with the Standard Protocol Items: Recommendations for Interventional Trials (SPIRIT) statement 2013 [41]. Figure 1 shows the schedule of enrolment, interventions and assessments according to the SPIRIT statement, and the SPIRIT checklist can be found in the electronic supplementary materials. Figure 2 provides an overview of the study procedures.

#### Recruitment

We will recruit eligible participants who have been referred to the clinic where the study is taking place. In addition, we will contact local primary/secondary care services, mental health services for children and young people and schools to advertise the trial and request referrals. We will also advertise the trial through social media, local newspapers and radio. Potential participants identified through these outreach activities will be asked to complete a screening form and indicate whether they would like treatment. If their responses indicate they may be eligible, the young person and their parent/carer will receive a telephone triage by the clinical team. If the triage indicates that they are likely to meet the inclusion criteria for the study, the young person will then be referred to the clinic. As is routine in the clinic, all potential participants will be invited to complete a diagnostic assessment to determine whether they meet diagnostic criteria for panic disorder and/or other anxiety disorders or depression. Where possible, the young person’s parent/carer will also undertake a diagnostic assessment reporting on their child’s difficulties. The young person and their parent/carer will be assessed separately. Assessments will be carried out by members of the team who are trained to reliability and will receive supervision for every assessment from a senior assessor with extensive experience of delivering and supervising diagnostic assessments and proven reliability. Assessments will be carried out by telephone or video call if face-to-face appointments are not possible. Adolescents and their parent/carer will also be asked to independently complete self-report questionnaires, reporting on the adolescent’s symptoms.

If the young person is eligible for the trial, they will be sent information leaflets (adolescent and parent/carer versions) and will then meet with a member of the research team to discuss the study further. If they agree to participate, written informed consent will then be given by the parent and the young person (or assent for young people under 16 years of age). Screening logs will be maintained for eligible participants not recruited, to inform acceptability of the study to young people. Reasons for non-participation in the trial will be collected anonymously.

#### Randomisation

Consenting participants will be randomised to receive individual sessions of brief cognitive therapy or standard CBT. To protect against bias, randomisation will be performed using an online randomisation system, *Sortition*. Participants will be randomised to receive either cognitive therapy or standard CBT, with a randomisation ratio of 1:1 and stratification based on the key baseline feature that is likely to be associated with outcome (i.e. panic disorder symptom severity). Participants will be randomised and informed of their allocation immediately following their consent to take part in the study and completion of baseline measures.

#### Treatment

Once randomised, participants will be allocated to a clinician for the relevant treatment arm. Clinicians delivering the trial interventions will be qualified therapists (e.g. children’s wellbeing practitioners, psychological wellbeing practitioners or clinical psychologists) and will only deliver treatment in one arm of the trial to prevent contamination. In line with good clinical practice [42], the young person will complete self-report measures prior to each treatment session to inform treatment.

#### Post-treatment and follow-up

As well as completing measures pre-treatment (and for young people, prior to each session), young people and their parents/carers will complete measures at the end of the main treatment sessions (post-treatment) and at 3-month follow-up. A further measure will be completed by young people 1 year following the end of treatment. The post-treatment and follow-up measures will be completed in the clinic or, if this is not possible, may be taken home and emailed or posted back to the clinic or completed online. At the 3-month follow-up assessment, participants will also have a further diagnostic assessment with an assessor who will not know which treatment they received. For participants who have discontinued with the treatment they were allocated to at randomisation, this follow-up assessment will be conducted at the time when it should have occurred had they continued in that treatment arm. Participants will be reimbursed with a voucher for their time and inconvenience in completing additional measures at pre- and post-treatment and follow-up assessments.

A subsample of young people and parents/carers will be invited to take part in a qualitative interview to discuss their experiences of treatment and the research study. We also aim to recruit key stakeholders involved in the research study. This will include service managers, clinicians involved in both treatment arms, referrers from a range of settings (e.g. schools and primary care) and professional backgrounds. Interviews will be conducted with participants individually and will be conducted by psychology students who have had training in qualitative research and will receive supervision from researchers with expertise in this approach. Interviews will take place either face to face, by video call or telephone call. A purposive sampling strategy will be adopted to identify participants who differ on demographic variables. For example, for the young people, this will include age, severity of panic disorder, level of impairment and the presence of comorbid difficulties at initial assessment, treatment outcomes and number of sessions attended. Participants who take part in the interviews will be reimbursed for their time and inconvenience in attending the interviews.

All data will be stored separately from other personally identifying data and will not be shared outside the research team to ensure participant confidentiality. Identifiable participant personal information will be stored on a secure, restricted access drive. All other data will be identified using a number, and the file with information linking numbers and names will be stored separately in a password-protected file on the university’s server.

#### Intervention

##### Brief cognitive therapy

This treatment was developed for the treatment of panic disorder by Professor David Clark and colleagues [43]. A variety of procedures described in a manual [44] will be used to reverse the maintaining factors identified in Clark’s (1985) cognitive model of panic disorder. These procedures will be introduced in four self-study modules. Prior to each of the first four sessions, the young person will read a self-study module and complete the written exercises and homework activities outlined in the module. These modules have been adapted to be suitable for adolescents, with PPIE input, e.g. changing the case studies from adults to young people, changes to the language and metaphors to be developmentally appropriate and adding graphics to make them visually appealing. Additional handouts dealing with common catastrophic thought (e.g. ‘I'll faint’ or ‘I’ll lose control’) may also be used if relevant to the young person’s concerns. In the session, the therapist will discuss the module with the young person and clarify any misunderstandings. The focus of the session will then be on experiential exercises in which bodily sensations and safety behaviours are systematically manipulated to demonstrate their adverse effects, as well as behavioural experiments in which the young person tests prespecified negative predictions while dropping their safety behaviours. Process measures will be used in each session to generate meaningful behavioural experiments. A habituation rationale will not be used, repeated exposure to the same situations not encouraged and the person will not be encouraged to develop and use positive self-talk before or during behavioural experiments. The treatment will involve five sessions of 30–90 min, with up to two 60-min booster sessions over the following 3 months.

Parents/carers will be encouraged to read the self-study modules to learn about panic disorder and cognitive therapy techniques and asked to support their child in completing the self-study module each week and carrying out behavioural experiments (or other relevant activities) between sessions. They will also be given a parent/carer self-study module, with advice on how they can support their child during the treatment. At the end of sessions, they will typically be involved in the relapse prevention plan, so that they can support their child with this after treatment ends. The therapist will also liaise with school at assessment and throughout therapy where necessary to support the young person in treatment. This may include providing the school with psychoeducation about panic disorder, cognitive therapy techniques and how school staff can support at different stages of the treatment (e.g. planning school-based behavioural experiments).

##### General CBT

Treatment will involve anxiety management techniques (e.g. psychoeducation about anxiety, breathing retraining and relaxation), before moving on to the development of an exposure hierarchy, in which the young person will develop, with their therapist, an ordered list of feared stimuli according to their anticipated fear reaction. They will then begin to face their fears by putting themselves in situations that they worry may trigger panic attacks, beginning with the least anxiety-proving situation, and learning that over time their anxiety diminishes. Once they can experience the situation without experiencing significant anxiety, they will then move on to the next situation on the list. Graded exposure is based on the idea that with repeated exposure to feared stimuli, the person will habituate and experience a reduction in fear. This leads them to learn a new set of associations and learn that they can cope. As is typical in routine clinical practice, clinicians will use worksheets that are freely available on the Internet to support the treatment. The scheduling and length of treatment sessions will be the same as the brief cognitive therapy treatment.

Parents/carers will be given psychoeducation about anxiety management and graded exposure. They will be asked to support their child outside session in carrying out exposure tasks and with the relapse prevention plan at the end of treatment. The therapist will also liaise with school at assessment and throughout therapy as necessary to support the young person in treatment.

### Measures and assessment

#### Screening questions

The screening questions can be found in the electronic supplementary materials. If potential participants respond yes to all items on the screening form, and indicate that they would like help, they will be invited to a telephone triage. Some discretion will be allowed, and therefore, young people who have responded ‘no’ to some items may still be invited to a telephone triage with a clinician to gain further information. If panic disorder is indicated, they will then receive a diagnostic assessment.

#### Demographic information

Demographic information will be collected from the parent on the pre-treatment questionnaire, and this will include information about the young person (age, gender and ethnicity) and the parent (relationship to young person, age, relationship status, education and employment). This will be used to describe the sample.

#### Diagnoses of anxiety disorders and comorbid disorders

A diagnostic assessment will be used to establish if the young person reaches diagnostic criteria for any anxiety disorders (including panic disorder) and mood disorders and to determine the primary presenting problem. A structured diagnostic interview will be carried out at baseline and 3-month follow-up. The anxiety section of the Anxiety Disorders Interview Schedule (ADIS-C/P [45];) will be used to determine whether the young person meets diagnostic criteria for any anxiety disorders, including panic disorder, and/or behavioural disorders, and to establish a clinician rating of severity for each disorder (CSR). The pre-treatment diagnosis with the highest CSR will be classed as the primary diagnosis. All final diagnoses and CSRs will be determined by consensus with a supervisor with proven reliability. Additionally, mood disorders will be assessed using the relevant sections of the Kiddie Schedule for Affective Disorders and Schizophrenia (K-SADS [46];), which is a structured diagnostic interview for DSM-IV affective disorders and schizophrenia.

#### Symptoms of panic, anxiety and depression

The Panic Disorder Severity Scale for Children and Adolescents (PDSS-A [47];) will be administered to assess change in the frequency and severity of adolescents’ panic disorder symptoms and anticipatory anxiety and associated agoraphobia, avoidance, fear, school/work and social impairments. There are seven items; each rated on a 0-4 scale, with a higher score indicated greater severity. It has been shown to have good psychometric properties with an adolescent population [47].

The Revised Child Anxiety and Depression Scale (RCADS [48]) will be used to measure symptoms of anxiety disorders and depression. This will be completed by the adolescent at every treatment session and by the adolescent and their parents/carers at pre, post, and 3-month follow-up appointments. The RCADS is a 47-item parent and child report scale which assesses symptoms of separation anxiety disorder, social anxiety disorder, generalised anxiety disorder, panic disorder, obsessive compulsive disorder and major depressive disorder. Responders rate how often each item applies on a scale of 0 (‘never’) to 3 (‘always’). The RCADS has been shown to have robust psychometric properties in children and young people from 7 to 18 years of age [49].

#### Functional impairment

The Child Anxiety Impact Scale (CAIS [50]) will be used to determine the extent to which anxiety interferes in the young person’s life. This will be completed at pre, post and 3-month follow-up appointments by young people and parents/carers. This measure covers three psychosocial domains (academic, social activities and home/family environments) and consists of 27 items rated on a 4-point scale from ‘not at all’ to ‘very much’. There are versions for children/adolescents and parents to complete, both of which have been shown to have good psychometric properties [50, 51].

The Clinical Global Impression Scale — Improvement (CGI-I [52];) will be used after the 3-month follow-up assessment to assess the young person’s post-treatment changes in global functioning. This requires the assessor to rate how improved the patient is compared to their initial assessment, prior to treatment, on a scale of 1 (‘very much improved’) to 7 (‘very much worse’). Final scores will be dichotomised to represent ‘much or very much improved’ versus ‘other’. A second rater will independently rate the CGI-I for all participants to establish inter-rater reliability.

#### Panic disorder-specific measures

Participants will complete panic disorder-specific measures to assess symptoms, cognitions and safety behaviours pre-treatment, post-treatment and at 3-month follow-up. The measures that have been designed for use with adults have been adapted for use with adolescents in this study based on consultation with service users. Consequently, their psychometric properties with adolescents have not yet been established. In the brief cognitive therapy treatment arm, participants will also complete the measures prior to each session, to guide the treatment sessions.

The Body Sensations Questionnaire [53] is a self-report measure of bodily symptoms relating to panic that an individual may experience and the level of fear they cause. This will be used to determine the degree to which participants are experiencing panic sensations. Respondents are asked to rate the level of fear they feel when experiencing 18 different bodily sensations, on a five-point scale from 1 (‘not at all’) to 5 (‘extremely’).

The Agoraphobia Cognitions Questionnaire [53], modified by Clark and colleagues [43] will be administered to measure cognitions associated with panic disorder. This measure includes 18 cognitions commonly associated with panic disorder. First, the respondent rates the frequency with which the thought occurred on a scale of 1 (‘thought never occurs’) to 5 (‘thought always occurs when I am anxious’). Second, the respondent rates the extent to which the thought was considered to be true on a scale of 0 (‘I do not believe this thought’) to 100 (‘I am completely convinced this thought is true’).

The Safety Behaviours Questionnaire [44] will be administered to identify panic-related safety-seeking behaviours. Adolescents will be asked to complete the self-report version. This involves 16 items, each rated on a four-point scale from ‘never’ to ‘always’.

The Mobility Inventory [54] is a validated measure of avoidance behaviour and associated panic attacks. Adolescents will be asked to complete the self-report version. Respondents are asked to rate how often they avoid situations both when they are on their own and when they are other people on a five-point rating scale (from ‘never’ to ‘always’) or ‘not applicable’.

The Panic Diary [44] is only given to participants in the cognitive therapy arm. It is used as part of the intervention (rather than an outcome measure) to record the frequency of panic attacks, identify triggers, sensations and thoughts, and to help the young person to answer their panic-related thoughts during an attack. At the beginning of therapy, patients only complete the left-hand side of the diary. As proficiency in answering panic-related thoughts develop, the whole diary is completed.

#### Session by session measures to guide treatment

The Outcome Rating Scale (ORS [55]) will be used to assess functioning across different areas of the young person’s life. It has four items: symptom distress, interpersonal wellbeing, social role and overall wellbeing. Each item is rated using a 10-centre visual analogue scale, with instructions to place a mark on each line. A higher score indicates better functioning. It has good reliability and validity with an adolescent population [56].

The Session Rating Scale (SRS [57, 58]) assesses key dimensions of an effective therapeutic relationship and is given at the end of each therapy session to get feedback from young people and parents/carers so that any issues related to therapeutic alliances can be immediately identified and addressed. It comprises four rating scales (relationship with the therapist, goals and topics, approach or method and an overall rating) and uses the same visual analogue scales as the ORS. It has well-established reliability and validity [58, 59].

The Goal-Based Outcomes tool [60] enables the young person to set up to three goals at the beginning of treatment as a way of evaluating their progress on a Goal Progress Chart. Progress towards individual goals is then periodically rated on a scale from 0 (‘no progress’) to 10 (‘goal has been reached’). Although this measure is now widely used in CAMHS, its psychometric properties have not yet been established.

#### Satisfaction with care

At the 3-month follow-up assessment, participants will rate their satisfaction with the service they have received using the Experience of Service Questionnaire (ESQ [61]), a measure that was developed by the Health Care Commission as a means of measuring satisfaction in Child and Adolescent Mental Health Services. There are versions for young people and their parents/carers to report on the extent to which they agree with 12 statements examining what the respondent liked about their care and the environment, what they felt needed improving and three free text sections for any other comments. It is routinely used within child and adolescent mental health services and has been demonstrated to have good psychometric properties [62].

#### Health economic measures

Health economic self-report questionnaires as detailed below are collected from parents/carers and young people at the pre, post and 3-month follow-up. Clinicians will use logs at each treatment and supervision session and any other times as required.

A societal perspective for costs will be adopted, and patient level resource use data will be collected from parents/carers on a Client Services Receipt Inventory (CSRI) using patient-health diaries to facilitate recall of healthcare resource use and from clinicians’ logs. This data will be provided by clinicians and parents/carers and will include all health and social care cost-generating resources (e.g. staff time for provision of treatment, training and supervision, GP use, referrals and other relevant services identified), non-NHS cost-generating services (e.g. educational services) and leisure and lost productivity time estimates for the parents/carers (e.g. days off school/college/work).

The EQ-5D (5L) [63] is a well-validated preference-based measure of health-related quality of life, designed to estimate quality-adjusted life years (QALYs), that is widely used across disease areas. The EQ-5D questionnaire contains five simple questions each concerned with a different domain of everyday life, i.e. mobility, self-care, usual activities, pain/discomfort and anxiety/depression. For each domain, the respondent must indicate whether he/she experiences no problems, slight problems, moderate problems, severe problems or extreme problems. The respondent’s answers provide a description or profile of the respondent’s quality of life, and a weight or value can then be placed on each profile using an existing UK tariff derived from the general public [63, 64]. The full questionnaire also includes a visual analogue scale (VAS) for participants to rate their overall health on a scale from 0 (‘worst imaginable health’) to 100 (‘best imaginable health’). Quality of life of carers will be assessed using the EQ-5D-5L self-report. The EQ-5D-Y [65, 66] was adapted directly from the EQ-5D to estimate utility values for young people (from 8 years). It covers the same domains as the EQ-5D, but the wording of the questions in each dimension is modified to make it appropriate to a younger age range. The EQ-5D (−Y; −5L) has established feasibility and reliability [63, 64].

The Child Health Utility 9D (CHU-9D [67, 68];) is a paediatric measure of health-related quality of life, which allows the calculation of QALYs for use in cost utility analysis. It includes nine dimensions (worried, sad, pain, tired, annoyed, schoolwork, sleep, daily routine, activities) each with five levels. The measure was originally developed with children aged 7–11 years and subsequently validated in an adolescent population (11–17 years) [68, 69]. The CHU-9D is also available in a ‘proxy’ version for parent/carer completion, and this will also be used.

#### Treatment credibility

Participant expectancies and views regarding treatment credibility will also be assessed prior to treatment through a credibility and expectation of improvement scale [33]. This consists of three items, rated on a scale from 0 (‘not at all’) to 10 (‘completely’), asking about how logical the treatment seems, confidence in its success at reducing their symptoms and their likelihood to recommend the therapy to a friend with similar symptoms.

#### Distinctness of therapies

To establish that the therapies in each arm are distinct from one another, a checklist of the components of each therapy will be given to therapists to complete at the end of every treatment session. The checklist has been designed for this trial and includes items that are distinct to either brief cognitive therapy (e.g. behavioural experiments designed to test out beliefs) or general CBT (e.g. development of a fear hierarchy). Therapists will indicate which components were carried out in the session that they have just completed. The ratings will be used to compare the content of the treatment sessions in the two arms to determine their distinctiveness.

#### Qualitative interviews

Semi-structured qualitative interviews will be conducted with the young people post-treatment with both groups to explore their experiences of treatment and the research trial, as well as parents/carers and stakeholders. Interviews will be one on one, conducted face to face, by video call or by phone, and will be recorded using digital audio recording (e.g. MP3) or through Microsoft Teams and then transcribed. Interviews will follow predetermined topic guides (see electronic supplementary materials). During interviews with young people about treatment, materials such as the participant’s therapy folder and flashcards of different core components of treatment may be used to assist the young person in talking about their experience and expressing their views about elements of treatment.

### Data analysis

A detailed statistical analysis plan will be developed and agreed with the Trial Steering Committee (TSC) before any analysis is carried out.

Analyses will investigate recruitment and retention rates presented as a CONSORT diagram providing both overall and individual arm results at all assessment points. Data completeness will be summarised for all clinical outcomes overall and for each study arm at each timepoint. Missing data will be explored to establish whether this is due to lack of response to specific questions, to the measure altogether or to loss of follow-up. Continuous clinical outcomes will be summarised with means and standard deviations and minimum and maximum values. Categorical outcomes will be summarised with numbers and proportions.

An exploratory comparison of between-group differences on the clinical outcome measures will be undertaken to assess whether the observed effect size is in line with the expected effect based on the literature, using analysis of covariance or a suitable alternative. Ninety-five per cent confidence intervals will be constructed for the between-group differences for each of the outcomes, adjusted for baseline, and compared with the literature. As this study is not powered to detect differences between arms, *p*-values will not be reported. Analysis of continuous clinical outcomes will follow a modified intention-to-treat (ITT) principle: all eligible, randomised patients will be included in the analysis. Per-protocol analyses will also be conducted, but these analyses will be treated as secondary to the ITT analysis.

The results of the feasibility trial will also be used to develop, refine and test a full statistical analysis plan for use in the subsequent main trial. The sample size for a future definitive trial will be based on an effect size that reflects the minimally important clinical difference between the two treatment arms [70, 71], based on previous literature, discussion with stakeholders, and qualitative and health economic data. The standard deviation obtained in the feasibility study for the likely outcome measure, the PDSS-A, will be used as an indication of the likely variance.

Suitability and acceptability of the economic measures will be assessed based on both rates of responses at the end of the feasibility study and from young peoples’ and their parents/carers’ feedback. Proportions of responses to healthcare resource use and health outcome measure questions will be presented separately for the two treatment arms at each assessment point. Missing data will be explored to establish whether this is due to lack of response to specific questions, to the measure altogether or to loss of follow-up. Rates of this missing data will also be compared to that of clinical measures to assess patterns in the response of certain participants. For both quality-of-life measures (i.e. the EQ-5D-Y and the CHU-9D), utility scores and quality-adjusted life years (QALYs) will be calculated and compared for both treatment groups to explore how sensitive each measure is to change over time. Finally, variation in quality of life as derived from the EQ-5D-5L will be reported and compared across both treatment groups.

For the qualitative data, thematic analysis [72] will be used to identify and describe emergent themes within the interviews about the experience of receiving treatment and taking part in the study. Thematic analysis has been used in previous studies to explore people’s experiences of psychotherapy (e.g. [73, 74]). This technique was chosen due to its flexible nature and because it is not associated with a particular theoretical framework [75]. A number of strategies will be employed to enhance the credibility and methodological rigour of the analysis [76], such as co-analysis of transcripts and use of reflexive practices in supervisory discussion.

### Trial and data monitoring

As this is a feasibility study being conducted at a single, secure site, the study investigators will be responsible for monitoring the conduct of the research, including data monitoring, managing adverse events and any decisions relating to early termination of the trial. Additionally, the trial management team, who will hold regular review meetings, will manage the safety and efficacy of the data. The trial will be overseen by a Trial Steering Committee, which will meet before the trial begins and then at key time points during the trial.

### Reporting of adverse events

All adverse events (between the time of consent to the study and the point at which the participant completes and submits the final set of follow-up questionnaires) will be recorded and reported. Follow-up of any adverse events by the research team will take place up until the point that appropriate procedures are completed.

## Discussion

The aim of the current study is to evaluate the feasibility of an RCT and to compare brief cognitive therapy to a general form of CBT for adolescents with panic disorder in the UK. This will be delivered largely by therapists, such as children’s wellbeing practitioners, trained to deliver brief evidence-based treatments and therefore able to provide cost-effective treatments within clinical services. The outputs from the study will provide a clear indication of the feasibility of a future definitive trial and, if indicated, the critical resources that will be required and key information to inform the design and maximise the successful completion of the trial. This has the potential to bring direct benefits to young people and their families, as well as services and society more broadly.

## Supplementary Information

**Additional file 1.** SPIRIT 2013 Checklist: recommended items to address in a clinical trial protocol and related documents.

## Data Availability

Anonymised datasets will be deposited in publicly available repositories (if available and appropriate) 1 year after the completion of the study.

## Abbreviations

ADIS-C/P: Anxiety Disorders Interview Schedule for Children and Parents
ACQ-adapted: Agoraphobia Cognitions Questionnaire (adapted)
BSQ-adapted: Body Sensations Questionnaire (adapted)
CAIS-C/P: Child Anxiety Impact Scale — child and parent version
CAMHS: Child and Adolescent Mental Health Service
CBT: Cognitive behaviour therapy
CGI-I: Clinical Global Impression — Improvement
CHU-9D: Child Health Utility (paediatric quality of life)
CONSORT: Consolidated Standards of Reporting Trials
CSRI: Client Services Receipt Inventory
DSM-5: Diagnostic and Statistical Manual of Mental Disorders, 5th edition
EQ-5D-Y: EuroQol (quality of life)
ESQ: Experience of Service Questionnaire
KSADS-C/P: Kiddie Schedule for Affective Disorders and Schizophrenia — child and parent version
MI-adapted: Mobility Inventory (adapted)
NHS: National Health Service
ORS: Outcome Rating Scale
PDSS: Panic Disorder Severity Scale for Adolescents
RCADS-C/P: Revised Child Anxiety and Depression Scale — child and parent versions
RCT: Randomised controlled trial
SBQ-adapted: Safety Behaviour Questionnaire (adapted)
SMFQ: Short Mood and Feelings Questionnaire
SPIRIT: Standard Protocol Items: Recommendations for Interventional Trials
SRS: Session Rating Scale
